# Editorial: Molecular mechanism and pharmacology of metabolism and cardiac remodeling

**DOI:** 10.3389/fphar.2023.1341004

**Published:** 2023-12-06

**Authors:** Mengya Chen, Saiyang Xie, Wei Deng

**Affiliations:** ^1^ Department of Cardiology, Renmin Hospital of Wuhan University, Wuhan, China; ^2^ Hubei Key Laboratory of Metabolic and Chronic Diseases, Wuhan, China

**Keywords:** metabolism, cardiac remodeling, heart failure, molecular mechanism, immunometabolism

The progress of Heart failure (HF) is associated with insufficient energy, substrate utilization disorders, and oxidative stress. In cardiac injuries, metabolic remodeling often precedes most changes (Lopaschuk et al., 2021). The Research Topic encompasses studies related to cardiac immunometabolism, mitochondrial metabolism, molecular biomarkers, special case report and drug development and treatment for heart failure.

Deviant mitophagy has been proved to induce energy metabolism dysfunction in the course of cardiac pathological events (Ranjbarvaziri et al., 2021). Zhou et al. discovered that mesaconine, a major cardiotonic component of Fuzi, significantly rescued cardiac dysfunction and impaired mitophagy in cardiomyocytes of mice administered doxorubicin. Furthermore, the role of mesaconine is mediated by the activation of PINK1-induced mitophagy. These results hold substantial significance for the development of clinical trials with mesaconine. Through pharmacology experiments in rhesus monkeys and rats, Zhang et al. demonstrated beneficial effects of QiShenYiQi Dripping Pills (QSYQ) on HF, which may be associated with pathways involved in myocardial energy metabolism. These findings establish a groundwork for the potential clinical application of QSYQ. Zhong et al. illustrated that traditional Chinese medicine QWQXI combined with western medicine therapy can enhance cardiac function in patients with chronic heart failure. QWQXI modulated inflammation mediated by glycerophospholipid and linoleic acid metabolism, thereby improving cardiac function in HF rats. This study provides a novel molecular foundation for investigating the intervention mechanisms of QWQXI. These findings offer new options for treating HF.

Immunometabolism plays an important role in the progression of cardiovascular remodeling (Williams et al., 2019). Meng et al. reviewed the latest researches on the immunometabolism and therapeutic strategies for atherosclerosis. The existence of immune cells and immune factors was related to atherosclerosis and atherosclerosis might be attenuated through the defensive immune response triggered by specific autoantigens or exogenous antigens. It reveals the potential of immunotherapy as a novel therapeutic strategy for atherosclerosis.

Zheng et al. demonstrated the possible mechanisms by which endogenous n-3 polyunsaturated fatty acids (n-3 PUFA) exerted protective effects against cardiac ischaemia-reperfusion (IR) injury. It was the first report that identified n-3 PUFA protected the heart by modulating the APLNR-dependent PI3K-AKT-mTOR signaling axis in the IR heart. These findings were further evidence that n-3 PUFA can protect mitochondrial structure and function. Previous studies have found a correlation between abnormal lipid metabolism and the progression of atrial fibrillation (AF) (Suffee et al., 2022). Utilizing ultra-high-performance lipidomics analysis, Huang et al. determined that phosphatidylethanolamine (PE) was correlated with AF. Further, they identified that PE may exacerbate Ang II-induced atrial fibrosis through inducing mitochondrial damage and ferroptosis in cardiomyocytes. This study suggested that inhibiting PE and ferroptosis may be a promising therapeutic strategy for AF.

Zou et al. presented a case report of idiopathic hypereosinophilic syndrome (IHES) complicated with intracardiac thrombus, which is a rare cardiac complication of HIS. Based on this case, novel oral anticoagulant (NOAC) therapy may be an effective option. This case contributed to a better comprehension of the pathology and cardiac complications of IHES.

In conclusion, the “Molecular Mechanism and Pharmacology of Metabolism and Cardiac Remodeling” Research Topic focuses on recent insights into the molecular mechanism and pharmacology in cardiac metabolic remodeling and novel therapeutic targets for cardiac remodeling and heart failure.

## References

[B1] Lopaschuk G. D. Karwi Q. G. Tian R. Wende A. R. Abel E. D. (2021). Cardiac energy metabolism in heart failure. Circ. Res. 128, 1487–1513. 10.1161/CIRCRESAHA.121.318241 33983836 PMC8136750

[B2] Ranjbarvaziri S. Kooiker K. B. Ellenberger M. Fajardo G. Zhao M. Vander Roest A. S. (2021). Altered cardiac energetics and mitochondrial dysfunction in hypertrophic cardiomyopathy. Circulation 144, 1714–1731. 10.1161/CIRCULATIONAHA.121.053575 34672721 PMC8608736

[B3] Suffee N. Baptista E. Piquereau J. Ponnaiah M. Doisne N. Ichou F. (2022). Impacts of a high-fat diet on the metabolic profile and the phenotype of atrial myocardium in mice. Cardiovasc Res. 118, 3126–3139. 10.1093/cvr/cvab367 34971360

[B4] Williams J. W. Huang L. H. Randolph G. J. (2019). Cytokine circuits in cardiovascular disease. Immunity 50, 941–954. 10.1016/j.immuni.2019.03.007 30995508 PMC6924925

